# Infections Caused by *Stenotrophomonas maltophilia* in Recipients of Hematopoietic Stem Cell Transplantation

**DOI:** 10.3389/fonc.2014.00232

**Published:** 2014-08-25

**Authors:** Khalid Ahmed Al-Anazi, Asma M. Al-Jasser

**Affiliations:** ^1^Section of Adult Hematology and Oncology, Department of Medicine, College of Medicine, King Khalid University Hospital, King Saud University, Riyadh, Saudi Arabia; ^2^Central Laboratory, Ministry of Health, Riyadh, Saudi Arabia

**Keywords:** *Stenotrophomonas maltophilia*, bacteremia, neutropenia, hematopoietic stem cell transplantation, drug resistance

## Abstract

*Stenotrophomonas maltophilia* (*S. maltophilia*) is a globally emerging Gram-negative bacillus that is widely spread in environment and hospital equipment. Recently, the incidence of infections caused by this organism has increased, particularly in patients with hematological malignancy and in recipients of hematopoietic stem cell transplantation (HSCT) having neutropenia, mucositis, diarrhea, central venous catheters or graft versus host disease and receiving intensive cytotoxic chemotherapy, immunosuppressive therapy, or broad-spectrum antibiotics. The spectrum of infections in HSCT recipients includes pneumonia, urinary tract and surgical site infection, peritonitis, bacteremia, septic shock, and infection of indwelling medical devices. The organism exhibits intrinsic resistance to many classes of antibiotics including carbapenems, aminoglycosides, most of the third-generation cephalosporins, and other β-lactams. Despite the increasingly reported drug resistance, trimethoprim-sulfamethoxazole is still the drug of choice. However, the organism is still susceptible to ticarcillin-clavulanic acid, tigecycline, fluoroquinolones, polymyxin-B, and rifampicin. Genetic factors play a significant role not only in evolution of drug resistance but also in virulence of the organism. The outcome of patients having *S. maltophilia* infections can be improved by: using various combinations of novel therapeutic agents and aerosolized aminoglycosides or colistin, prompt administration of *in vitro* active antibiotics, removal of possible sources of infection such as infected indwelling intravascular catheters, and application of strict infection control measures.

## Introduction

*Stenotrophomonas maltophilia* (*S. maltophilia*) is a free living, motile, aerobic, oxidase negative, glucose non-fermentative Gram-negative bacillus (GNB). It is frequently isolated from water, soil, animals, plants, and hospital equipment (1–5). *S. maltophilia* is the only species of the genus *Stenotrophomonas* known to infect humans. The organism was first isolated from pleural fluid by Edwards in 1943 and it was called *Bacterium brookeri* (1, 3). Hugh and Ryschenkow reclassified *Bacterium brookeri* as *Pseudomonas maltophilia* in 1961. Twenty years later, Swings et al. proposed reclassification of *P. maltophilia* as *Xanthomonas maltophilia*. In the year 1993, the genus *Stenotrophomonas* was proposed by Palleroni and Bradbury, so the organism was finally named as *S. maltophilia* (1). Risk factors for *S. maltophilia* infections in the general population are very variable and they are listed in Table 1 (1–14).

**Table 1:** **Risk factors for *S. maltophilia* infections in the general population**.

(1) Malignancy, particularly hematological malignancy (2) Human immunodeficiency virus (HIV) (3) Cystic fibrosis (4) Intravenous drug abuse (5) Surgical and accidental trauma (6) Prolonged hospitalization (7) Admission to ICU and mechanical ventilation (8) Indwelling vascular catheters and urinary catheters (9) Corticosteroids and immunosuppressive therapy (10) Prior treatment with broad-spectrum antibiotics (11) Gastrointestinal tract colonization and mucositis (12) Hematopoietic stem cell transplantation (HSCT) (13) Travel to hospital by air

*ICU, intensive care unit*.

## Infections Caused by *S. maltophilia*

*S. maltophilia* causes various infectious complications in immunocompromised individuals and these include bacteremia, endocarditis, respiratory tract infections, meningitis, urinary tract infections, skin and soft tissue infections, mastoiditis, bone and joint infections, peritonitis, typhlitis and biliary sepsis, wound infections, and central venous catheter (CVC)-related infections (1–3, 5–7, 11, 15–17). Occasionally, distinguishing between colonization and infection can be problematic (1–3). *S. maltophilia* infections can be complicated by septic shock, respiratory failure, pulmonary hemorrhage, metastatic cellulitis, tissue necrosis that may be extensive, septic thrombophlebitis, disseminated infection, and death (5–8, 11).

## *S. maltophilia* Infections in Recipients of HSCT

Recipients of various forms of hematopoietic stem cell transplantation (HSCT) are at risk of a wide range of infectious complications due to their severely suppressed immunity. Infections caused by *S. maltophilia* cause significant morbidity and mortality in this group of patients (2, 5, 7, 9, 10, 13, 14). Peculiar risk factors predispose recipients of HSCT to *S. maltophilia* infections and these are included in Table 2 (2, 5, 7, 9, 10, 13, 14). The most commonly encountered infectious complications related to *S. maltophilia* are pneumonia, bacteremia, and CVC-related infections. *S. maltophilia* may coexist with other infections caused by bacterial, viral, fungal, and protozoal agents (5, 6).

**Table 2:** **Risk factors for *S. maltophilia* infections in HSCT recipients**.

(1) Underlying disease being a hematological malignancy: leukemia, lymphoma, or multiple myeloma (2) Cytotoxic chemotherapy (3) Radiotherapy (4) Neutropenia and bone marrow aplasia (5) Graft versus host disease (GVHD) (6) Immunosuppressive therapies: corticosteroids and cyclosporine-A (7) Monoclonal antibodies (8) Indwelling vascular catheters and urinary catheters (9) Prior treatment with broad-spectrum antibiotics (10) Prolonged hospitalization (11) Admission to ICU and mechanical ventilation (12) Gastrointestinal tract colonization and diarrhea (13) Severe mucositis

*ICU, intensive care unit*.

*S. maltophilia* infections have been described in various forms of HSCT: autologous transplant and allogeneic, sibling related, matched unrelated donor and umbilical cord blood, HSCT (5, 7–9, 13, 14).

## CVC Infections Caused by *S. maltophilia*

Central venous catheter-related infections are the most frequent cause of hospital-acquired bacteremia in critically ill patients. Over the past two decades, the incidence of CVC-related bacteremia due to GNB has increased. Among non-fermentative GNB, *S. maltophilia* is the most frequently isolated pathogen (18). In patients with cancer, hematological malignancy (HM), and in recipients of HSCT acquiring bacteremia, CVC-related infection should be considered seriously as *S. maltophilia* infections are commonly associated with the presence of CVCs in these immunocompromised hosts (13, 19, 20). *S. maltophilia* CVC-related infections have also been reported in HSCT recipients. These infections cause bacteremia that may be recurrent, may occur as part of polymicrobial infection, and carry high mortality rates (2, 5, 13, 14).

Indications for removal of CVCs include: (1) bloodstream infection (BSI) due to GNB particularly multidrug-resistant (MDR) isolates, (2) critically ill patients such as high risk immunocompromised hosts including patients with HM and recipients of HSCT, and (3) presence of complications such as: severe sepsis and septic shock, tunnel infection, suppurative thrombophlebitis, and infective endocarditis (21). Administration of appropriate antibiotic therapy, immediate removal of infected catheters, and implementation of strict hand hygiene for health-care personnel are crucial in the management of CVC-associated infections and are associated with good prognosis (2, 5, 13, 14, 19, 20).

In patients with documented CVC-related GNB infections, removal of CVC should be performed within 48–72 h (20, 22). However, some studies have reported that, even in patients with long-term CVC having infections caused by GNB, catheter salvage can be achieved in 70% of cases by antimicrobial therapy and decontamination of the catheter lock with an anti-infective lock solution (23). Novel securement devices and antibiotic lock solutions have also been shown to reduce the risk of intravascular device-related BSIs in prospective randomized trials (24). Other studies have shown that, despite the use of appropriate antibiotics, keeping an infected catheter *in situ* causes not only recurrence in infection by the same GNB or other micro-organisms but also death (20, 22). Recurrence of CVC-related infections has been reported after long latency period reaching 200 days (20).

## Pneumonia Caused by *S. maltophilia*

In hospitalized HSCT recipients, the respiratory tract is the most common site of isolation of the organism (2, 3). In recipients of HSCT, the risk factors for hospital-acquired pneumonia due to *S. maltophilia* include: (1) immunocompromised health status, (2) indwelling CVCs, (3) mechanical ventilation, (4) exposure to and duration of broad-spectrum antibiotic therapy, (5) prolonged hospitalization, (6) admission to an intensive care unit (ICU), and (7) presence of underlying lung disease such as chronic obstructive airway disease (25, 26). Treatment of ventilator-associated pneumonia (VAP) caused by *S. maltophilia* includes: (1) high dose trimethoprim-sulfamethoxazole (TMP-SMZ), which is still considered the drug of choice based on its excellent *in vitro* activity, and (2) an alternative antimicrobial therapy in patients having drug resistance, allergy, or adverse effects related to TMP-SMZ that includes a new fluoroquinolone or a combination of doxycycline and aerosolized colistin (27, 28). Despite the recent improvement in the short-term prognosis of HSCT recipients transferred to ICU, *S. maltophilia*-associated VAP and bacteremia still carry significant morbidity and mortality (28, 29).

In patients with HM and in recipients of HSCT having severe lung infection caused by *S. maltophilia*, life-threatening pulmonary alveolar hemorrhage (PAH) causing acute respiratory failure has been reported (7, 8, 30–32). The risk factors for PAH in these patients include prolonged cytopenias and high C-reactive protein levels at the onset of BSI or pneumonia (7, 8). However, the mechanism of *S. maltophilia*-induced PAH remains unclear (7, 8). Despite the expected dismal outcome, prompt institution of TMP-SMZ in combination with other antimicrobials, provision of advanced respiratory care, and adequate blood products including granulocyte transfusions, if needed, may be life-saving in these immunocompromised individuals (7, 8, 30–32). In a study that included 223 recipients of HSCT who had diffuse pulmonary infiltration with respiratory compromise: 39% of patients had PAH and 43% of patients had an organism cultured from BAL. Patient who had PAH and an organism isolated from BAL had the worst outcome and isolation of a microbial organism from BAL was a strong predictor of poor prognosis (31).

## Bacteremia Caused by *S. maltophilia*

*S. maltophilia* bacteremia is hospital-acquired in 76% of patients and has been reported in up to 24% of recipients of allogeneic HSCT (9, 18). The risk factors for *S. maltophilia* bacteremia are included in Table 3 (5, 6, 9, 12, 14, 18, 19, 33–36). The sources of infection in patients having *S. maltophilia* bacteremia include: primary bacteremia with no known source, CVC-associated infections, respiratory tract infections such as pneumonia in mechanically ventilated patients, gastrointestinal tract (GIT) infections, and faulty replacement of hand washing soap (5, 6, 9, 10, 14). Outbreaks of *S. maltophilia* bacteremia have been described in recipients of allogeneic HSCT having neutropenia and mechanical ventilation (9, 10). These outbreaks were attributed to: (1) hospital equipments acting as reservoirs for *S. maltophilia*, (2) prevalence of GIT colonization with the organism in patients who develop invasive infections, (3) selective pressure of antimicrobial therapy, and (4) faulty replacement of hand washing soap (9, 10).

**Table 3:** **Risk factors for *S. maltophilia* bacteremia**.

(1) CVC infection (2) Prolonged hospitalization (3) Admission to ICU and mechanical ventilation (4) Prior treatment with broad-spectrum antibiotics (5) Severe neutropenia (6) Corticosteroid treatment (7) Underlying disease being a hematological malignancy (8) Aggressive cytotoxic chemotherapy (9) Immunosuppressive therapy (10) Major trauma or recent surgical intervention (11) Severe mucositis (12) Total parenteral nutrition

*ICU, intensive care unit; CVC, central venous catheter*.

The overall mortality related to *S. maltophilia* bacteremia may reach 33% (5, 18). Studies have also shown that 14-day and 30-day mortality rates in patients having *S. maltophilia* BSIs are 24 and 35%, respectively (6, 37). The 30-day mortality rate related to *S. maltophilia* bacteremia is higher and has worse prognosis compared with bacteremia related to other glucose non-fermenting GNB such as *Pseudomonas aeruginosa* and *Acinetobacter* species (37). Factors independently associated with mortality in patients having *S. maltophilia* bacteremia include: (1) profound neutropenia, (2) thrombocytopenia, (3) septic shock or hypotension at the onset of bacteremia, (4) inappropriate antimicrobial therapy, and (5) having high sepsis-related organ failure assessment index (6, 14, 18). In patients having *S. maltophilia* BSIs, good prognosis is associated with early administration of appropriate antimicrobial therapy, removal of infected CVCs, recovery of bone marrow function, and taking enough preventive and isolation measures (5, 14, 35, 36).

## Antimicrobial Susceptibility and Therapeutic Options

*S. maltophilia* has been shown to be susceptible to the following antibiotics: (1) TMP-SMZ, (2) ticarcillin-clavulanate, (3) piperacillin-tazobactam, (4) cephalosporins such as ceftazidime, (5) aminoglycosides such as streptomycin and amikacin, (6) tetracyclines such as minocycline, doxycycline, and tigecycline, (7) fluoroquinolones such as ciprofloxacin, levofloxacin, moxifloxacin, gatifloxacin, and trovafloxacin, (8) polymyxins such as colistin sulfate, and (9) other antibiotics such as rifampicin, chloramphenicol, salbactam-cefoperazone, moxalactam as well as silver sulfadiazine (1, 3, 5–7, 10, 11, 14, 15, 38–48).

The organism has been reported to be resistant to the following antimicrobials: (1) carbapenems such as imipenem and meropenem, (2) amoxicillin-clavulanic acid, (3) aminoglycosides such as tobramycin and gentamicin, (4) most of the cephalosporins such as cefpirome and cefsulodin, (5) quinolones and most of the anti-pseudomonal penicillins. Also, some strains have been reported to be resistant to tetracyclines, ciprofloxacin, chloramphenicol (1, 5, 10, 11, 16, 39, 49–51).

In a large survey (CANWARD 2007-11 Study) performed in Canada between 2007 and 2001 that included 22,746 clinical isolates from hospitals in 8 districts: *S. maltophilia* accounted for 1.4% of all isolates and the organism ranked number 16 among the 20 most common pathogens isolated from Canadian hospitals (42). Also, in a 6-month prospective multicenter study performed in 9 teaching hospitals in France that included 158 patients, *S. maltophilia* was the most common non-fermentative GNB (39%) isolated from hospitalized patients (52). In the former study, tigecycline displayed good *in vitro* activity against MDR isolates of *S. maltophilia* and in the latter study the high incidence of MDR among *S. maltophilia* isolates rendered empirical antimicrobial therapy inappropriate (42, 52).

Various studies have shown synergistic activity when the following combinations are used: TMP-SMZ and ticarcillin-clavulanate, TMP-SMZ and ceftazidime, ticarcillin-clavulanate and levofloxacin, ticarcillin-clavulanate and aztreonam as well as ceftazidime and ciprofloxacin (43–45). Only one study showed that TMP-SMZ and fluoroquinolones are equally effective once used as monotherapies in treating *S. maltophilia*-related infections (53). MDR isolates of *S. maltophilia* have shown excellent responses to combinations of new fluoroquinolones and tetracycline derivatives (46). Future therapies that have the potential to slow the development of drug resistance include phage therapy, nanoemulsions, use of antisense RNA to target genes involved in virulence, and use of adjuvants that restore antibiotic efficacy (43).

Studies on *in vitro* susceptibility of *S. maltophilia* performed in several countries have shown variable results (42, 47, 48, 52–56). A Greek 6-year survey showed that *in vitro* susceptibility of *S. maltophilia* to TMP-SMZ, colistin, netlimicin, and ciprofloxacin were 85.3, 91.2, 85.3, and 82.4%, respectively (47). A Spanish survey showed: (1) *S. maltophilia* was found to be highly resistant to several antibiotics, and (2) TMP-SMZ and ticarcillin-clavulanate were the only antimicrobials that exhibited good *in vitro* activity against *S. maltophilia* (48). One Korean study showed that susceptibility rates of *S. maltophilia* to TMP-SMZ, minocycline, and levofloxacin were 96, 99, and 64% respectively (54). Another Korean survey showed: (1) the degree of antimicrobial resistance, even to TMP-SMZ, varied considerably between isolates, and (2) accurate identification was essential for appropriate selection of treatment options and alternative therapies in case of TMP-SMZ resistance (55). Finally, a study performed in Saudi Arabia showed that *in vitro* resistance rates of *S. maltophilia* to various antibiotics were as follows: imipenem 100%, gentamicin 87.4%, aztreonam 86%, ciprofloxacin 77%, piperacillin–tazobactam 61%, ceftazidime 57%, and TMP-SMZ 9.5% (56).

## Drug Resistance in *S. maltophilia*

Unfortunately, the organism is resistant to several antimicrobials and there is a growing concern about the magnitude of drug resistance displayed by *S. maltophilia* (2–4, 17, 40, 51, 57–63). There are several mechanisms of drug resistance and these are listed in Table 4. Genetic determinants of drug resistance in *S. maltophilia* include class 1 integrons carrying sul_1_ gene and insertion sequence common region (ISCR) elements carrying sul_2_ gene (Table 5) (2–4, 17, 40, 51, 57–63). The mechanisms of drug resistance vary from one antimicrobial to another and at times each antibiotic may have different enzymes or genes involved in the evolution of its drug resistance. For example: class 1 integrons (sul_1_) and ISCR elements (sul 2) are involved in the resistance to TMP-SMZ, sme DEF, and smQnr are involved in quinolone resistance and three different mechanisms, aac (6′) Iz acetyl transferase modifying enzyme, efflux pumps, and outer membrane modifications, are involved in drug resistance to aminoglycosides (Tables 4 and 5) (51, 57, 58, 62).

**Table 4:** **Mechanisms of *S. maltophilia* drug resistance**.

Mechanism	Examples
(1) Modification of outer membrane proteins	Protein expression Reduction in permeability
(2) Expression of chromosomally encoded multidrug efflux pumps	Sme ABC Sme DEF Cadmium efflux determinants: Cad A and Cad C Resistance-nodulation cell division (RND) efflux pump.
(3) Enzyamatic mechanisms	Expression of chromosomal or plasmid encoded β-lactamases such as L_1_ and L_2_ β-lactamases Aminoglycoside acetylcholine modifying enzyme a (6′) Iz gene. Inactivation of macrolide phosphotransferase.
(4) Target site alterations	Phosphoglucomutase gene mutations on liposaccharide. Mutations of bacterial topoisomerase and gyrase genes.

**Table 5:** **Genetic determinants of drug resistance**.

Genetic determinant	Antibiotics involved in drug resistance
Sul 1 and Sul 2	Trimethoprim–sulfamethoxazole
Sm Qnr R	Quinolones, tetracyclines.
ISCR	Trimethoprim–sulfamethoxazole, quinolones, aminoglycosides, β-lactams
Sme ABC	Quinolones such as ciprofloxacin, aminologycosides, β-lactams
Sme DEF	Quinolones such as ciprofloxacin and ofloxacin Macrolides Chloramphenicol Tetracycline Novobiocin
Sme IJK	Quinolones such as ciprofloxacin and levofloxacin Aminoglycosides Tetracyclines
Sme OP and Tol C sm	Aminoglycosides such as gentamicin and amikacin Trimethoprim-sulfamethoxazole Nalidixic acid Chloramphenicol Erythromycin and other macrolides Doxycycline Leucomycin Chemicals such as tetrachlorosalicylanilide.
Sme Z	Aminoglycosides

Several molecular mechanisms contribute to MDR and they include plasmids, integrons, and transposons (2). Resistance of *S. maltophilia* is mediated by a relatively impermeable cell membrane, the production of β-lactamases (L_1_ and L_2_), and an efficient resistance nodulation cell division (RND) efflux pump Sme DEF able to extrude fluoroquinolones, chloramphenicol, and tetracycline (64). Biofilm formation leads to colonization of mucosal surfaces, wounds, fluids, catheters, and implants and this contributes to persistence of the organism (2, 65, 66). Persistence of *S. maltophilia*, virulence of the bacterium, and inappropriate use of broad-spectrum antibiotics predispose to drug resistance (2).

Studies have shown that genetic factors play a major role in drug resistance (2, 67). The wide range of antimicrobial drug resistance genes and mobile genetic elements found suggests that *S. maltophilia* can act as a reservoir of antimicrobial drug resistance determinants in a clinical environment, which is an issue of considerable concern (67). The genome sequence of *S. maltophilia* reveals its capacity for environmental adaptation that presumably contributes to its persistence *in vivo* (67). The genome sequencing of *S. maltophilia* isolates K 279a and AU 12-09 revealed huge number of genes coding for MDR to several antimicrobials (67, 68). Although it is not a highly virulent organism, the large number of genes involved, the MDR phenotype, and its ability to attach to mucosal surfaces and surfaces of hospital equipment make the organism more persistent and more difficult to eradicate (67).

*S. maltophilia* has high intrinsic resistance to a variety of structurally unrelated antimicrobials including aminoglycosides, quinolones, and β-lactams. Multidrug efflux transport systems are an important mechanism for bacteria to combat antimicrobial agents. Among efflux transport systems, the RND-type multidrug efflux systems play a critical role in MDR, particularly in GNB (69). Two efflux systems have been identified in *S. maltophilia*, Sme ABC and Sme DEF. Genome sequence analysis has revealed that *S. maltophilia* K279a encodes many putative RND efflux systems including Sme ABC, Sme DEF, Sme GH, Sme IJK, Sme MN, Sme OP, Sme VWX, and Sme YZ. Sme U_1_–V–W–U_2_–Y, which is a novel RND-type efflux pump operon, composed of five genes, has been characterized (69). The SmQnr protein encoded by the chromosome qnr determinant of *S. maltophilia* has been shown to contribute to the intrinsic quinolone resistance of *S. maltophilia*. Inactivation of SmqnrR contributes to an acquired increase in quinolone and tetracycline minimum inhibitory concentrations (MICs) for *S. maltophilia* (Table 5) (70).

A five-gene cluster (tolCsm, Sme RO, Sme D, Sme P, and pcm) of *S. maltophilia* was characterized (71). The pcm-tolCsm makes a significant contribution to multidrug resistance of *S. maltophilia* (72). Deletion of tolCsm increases susceptibility of *S. maltophilia* KJ_2_ to: (1) several antimicrobial agents including aminoglycosides, macrolides, doxycycline, nalidixic acid, chloramphenicol, and TMP-SMZ, and (2) chemical compounds including carbonylcyanide 3- chlorophenyl hydrazine, tetrachlorosalicylanilide, fusidic acid, paraquat, crystal violet, menadine, and plumbagin (72). The sul_1_ and sul 2 genes lead to a high rate of TMP-SMZ resistance (Table 5) (73). ISCR elements are associated with a variety of antibiotic resistance genes. At least 13 ISCRs have been identified and they are associated with resistance to TMP-SMZ, quinolones, aminoglycosides, and β-lactams (74).

The increasing number of *S. maltophilia* isolates seen in ICUs, their resistance to mainstay antibiotics, their genetically diverse nature and possible cross-transmission within hospitals strongly underscore the need for continuous surveillance for *S. maltophilia* in hospital settings (75). Environmental isolates of *S. maltophilia* have been found to have lower overall mutation frequencies compared with clinical isolates (76). Trichloroethylene (TCE) is one of the major ground water pollutants throughout the world (77). *S. maltophilia* PM102 is capable of growth on TCE as the sole carbon source and has the capacity to degrade TCE efficiently (77, 78). *S. maltophilia* PM102 has been identified by 16S rDNA sequencing and has been characterized by Fourier transform infrared spectroscopy (FT-IR) (78, 79). Triclosan is one of the most widely used biocides. Binding of triclosan to Sme T induces antibiotic resistance in *S. maltophilia*. Triclosan is a good inducer of the expression of MDR efflux pump Sme DEF and exposure of *S. maltophilia* to triclosan selects for antibiotic resistance (80).

## New Diagnostic Methods

As infections caused by *S. maltophilia* are associated with considerable morbidity and mortality and as the organism has the potential to acquire resistance to many classes of antimicrobials, it is essential to make diagnosis and identification of the organism as early as possible in order to institute appropriate antibiotic therapy promptly. This can be achieved by utilizing the new or advanced laboratory and radiological diagnostic tools (81).

### Molecular diagnostics in *S. maltophilia* infections

Detection of *S. maltophilia* in blood cultures can be achieved more rapidly by combining flow cytometry and fluorescence *in situ* hybridization compared to standard detection methods (81). Both conventional and real-time polymerase chain reaction (PCR) are sensitive methods for diagnosing GNB infections including those caused by *S. maltophilia* in cancer patients having febrile neutropenia. Although PCR is more costly than routine blood cultures, its use in patients with HM having febrile neutropenia can be justified as studies have shown that the use of PCR under such circumstances has improved the outcome and has reduced the mortality related to these serious infections (81–83).

Early identification of pathogens and antimicrobial resistance in BSIs decreases morbidity and mortality in patients with HM and in recipients of HSCT (83–85). Molecular techniques, such as PCR, ribosomal RNA, and nucleic acid amplification, can augment cultural methods in the diagnosis of causal agents of bacteremia in these patients so that appropriate antimicrobial therapies can be commenced particularly in culture-negative infection episodes (83, 85). Although blood cultures remain the gold standard for detecting BSIs, real-time PCR is a valuable complementary tool in the management of BSIs in recipients of HSCT, thus allowing early identification of pathogens and antimicrobial resistance genes (84).

The LightCycler SeptiFast assay, a multiple blood PCR technique, when combined with blood cultures provides clinically relevant information for the diagnosis of blood culture-negative febrile neutropenia in patients with persistent fever despite antibacterial therapy or when a non-responding bacterial infection or an invasive fungal infection is suspected (86, 87). Results of the SeptiFast assay may lead to a more targeted antimicrobial treatment early after the onset of fever (88).

### Diagnostics in pulmonary complications of *S. maltophilia*

In patients with febrile neutropenia, computed axial tomography (CAT) scan of the thorax is more sensitive than chest X-ray (CXR) in detecting pulmonary infections (89). Positron emission tomography scans are also valuable in defining the extent of infection and guiding management in immunocompromised individuals having febrile neutropenia (90).

In febrile neutropenic patients, having a normal CXR does not rule out pneumonia. High resolution CAT (HR-CAT) scans can detect pneumonia 5 days earlier than CXR (91, 92). Therefore, patients with febrile neutropenia with no evidence of pneumonia on CXR should have HR-CAT scans (91, 92). The most common HR-CAT scan findings in recipients of HSCT having pneumonia are air-space consolidation, micronodular shadows, and ground-glass opacities involving middle and lower lung fields (93). A study that included 112 patients with HM and recipients of HSCT having febrile neutropenia showed the following results: sensitivity of HR-CAT scans in detecting pneumonia in patients with febrile neutropenia and in recipients of HSCT was 87 and 88%, specificity was 57 and 67%, respectively, while negative predictive values were 88 and 97%, respectively (92).

Fiber-optic bronchoscopy and bronchoalveolar lavage (BAL) have generally been considered as safe and accurate procedures for evaluation of patients having pulmonary infiltrates (94–96). The diagnostic yield of bronchoscopy in HSCT recipients may reach 75% and the results of BAL may change treatment in up to 51% of episodes of pneumonia (94–96).

Lung biopsies carry significant risk of morbidity and mortality in recipients of HSCT who are fragile, thrombocytopenic, and immunocompromised (94). Patients with HM and recipients of HSCT having pulmonary infiltrates or nodular lung lesions are at risk of treatment failure once conventional broad-spectrum antimicrobial therapies are used on empirical basis as filamentous fungi and MDR-GNB such as *Pseudomonas aeruginosa* and *S. maltophilia* may be involved etiologically (97). In these patients, pulmonary lesions should be characterized by HR-CAT scans, bronchoscopy, BAL, and even lung biopsies, if absolutely indicated (98). The choice of optimal biopsy should be individualized and guided by several patient and institutional factors. It is highly recommended to adopt interdisciplinary approach in order to optimize diagnosis and treatment (98).

In patients with HM and in recipients of HSCT, molecular diagnostic techniques such as PCR and nucleic acid amplification are promising tool for the rapid etiological diagnosis of pneumonia (99, 100). Their usefulness in the early diagnosis of viral and atypical pneumonia is well documented. However, their role in early detection of bacterial causes of pneumonia in patients with HM and in recipients of HSCT needs further evaluation and standardization of the diagnostic tools utilized (99, 100).

## Treatment of *S. maltophilia* Infections

Trimethoprim-sulfamethoxazole is still the drug of choice for *S. maltophilia* infections (Table 6) (4, 5, 14, 16, 67, 101, 102). Unfortunately, resistance to TMP-SMZ is increasingly reported and the rate of resistance is 8.3–9.1% (4, 16, 34, 36, 101, 102). However, for successful eradication of *S. maltophilia* infections, TMP-SMZ must be given in a sufficient dose and at a proper frequency in order to produce adequate concentrations at the site(s) of infection. Determination of the appropriate dosing regimen requires optimum knowledge and utilization of the pharmacokinetics and pharmacodynamics of TMP-SMZ (103). Currently, the most effective antibiotics against *S. maltophilia* are: TMP-SMZ, ticarcillin-clavulanic acid, minocycline, and the new fluoroquinolones such as moxifloxacin, ofloxacin, and levofloxacin. These agents can be used either alone or in various combinations (36, 64).

**Table 6:** **This shows available and future therapeutic modalities for *S. maltophilia* infections**.

Therapeutic modality	Examples and details
Monotherapies	TMP-SMZ: still the drug of choice Fluoroquinolones: ciprofloxacin, levofloxacin, moxifloxacin, gatifloxacin, trovafloxacin, ofloxacin. Ticarcillin-clavulanate Tetracyclines: minocycline, tigecycline Cephalosporines: ceftazidime.
Combination therapies	TMP-SMZ and ticarcillin-clavulanate TMP-SMZ and ceftazidime TMP-SMZ and tigecycline Tigecycline and amikacin Colistin and tigecycline Aerosolized colistin and doxycycline **MDR species:** colistin and rifampicin or colistin and TMP-SMZ
Targeted and future therapies	Inhibitors of efflux pumps Inhibitors of β-lactamases Antimicrobial peptides Cationic compounds Bacteriophages Nanoemulsions Telavancin and colistin Plant oils and constituents of green tea

*TMP-SMZ, trimethoprim-sulfamethoxazole; MDR, multidrug resistance*.

Alternative therapies to TMP-SMZ include: (1) monotherapies including cephalosporins such as ceftazidime; fluoroquinolones such as ciprofloxacin, levofloxacin including the aerosolized form, moxifloxacin, gatifloxacin, and trovafloxacin; ticarcillin-clavulanate and tetracyclines such as tigecycline or minocycline and (2) drug combinations such as: TMP-SMZ and ticarcillin-clavulanate, TMP-SMZ and tigecycline, tigecycline and amikacin, colistin and tigecycline, telavancin and colistin, or aerosolized colistin and doxycycline (Table 6) (2, 4, 17, 101, 104, 105). Several recent studies have shown the efficacy of levofloxacin monotherapy as an alternative to treatment with TMP-SMZ (37, 53, 106). Antimicrobials that are active against *S. maltophilia* should preferably be used in combinations, particularly once MDR *S. maltophilia* is isolated (42–48). In the treatment of infections caused by MDR *S. maltophilia*, colistin can be used in combination with rifampicin or TMP-SMZ (107).

The Fourth European Conference on Infections in Leukemia (ECIL-4, 2011) recommended the following targeted therapies for *S. maltophilia* infections in patients with leukemia and in recipients of HSCT: (1) TMP-SMZ, (2) fluoroquinolones such as ciprofloxacin and moxifloxacin, (3) ticarcillin-clavulanate, and (4) in seriously ill or neutropenic patients, combination therapy may be considered such as TMP-SMZ and ceftazidime or TMP-SMZ and ticarcillin-clavulanate (41). Targeted therapy against MDR bacteria should be based on: (1) *in vitro* susceptibility data, (2) knowledge of the best treatment option against the particular species or phenotype of bacteria, (3) pharmacokinetic and pharmacodynamic data, and (4) careful assessment of risk–benefit balance (41). For infections due to GNB, antimicrobials should be preferably used in combination with other agents that remain active *in vitro* because of suboptimal efficacy in case of tigecycline and the risk of emergent resistance in case of fosfomycin. There is a growing problem of antimicrobial resistance among pathogens isolated from patients with HM and recipients of HSCT in many centers and this increasingly influences the choice of empirical antimicrobial therapy (41).

The standard therapy with TMP-SMZ may be difficult to administer in recipients of HSCT particularly those having cytopenias due to its myelosuppressive effects (14). In such patients, the following alternative antimicrobials can be used alone or in combinations: ceftazidime or ceftriaxone, ticarcillin-clavulanic acid, and levofloxacin or moxifloxacin (5, 14). An additional concern is that these patients receive plenty of other drugs such as cyclosporine-A, mycophenolate mofetil, triazoles, and antivirals that may predispose not only to more adverse effects but also to drug interactions (41). Therefore, use of specific antimicrobials should be cautiously considered. Antimicrobial stewardship aiming to minimize unnecessary broad-spectrum antibiotic use and its associated collateral damage and resistance selection is crucial in the current era of growing drug resistance (41). In patients with HM and in recipients of HSCT having infections caused by MDR *S. maltophilia*, IV colistin with dosage adjusted to renal function is relatively safe, even if given concomitantly with other nephrotoxic medications (108).

In patients with device-associated infections caused by *S. maltophilia*, fluoroquinolones particularly levofloxacin can be used to eradicate such infections once used in combination with other antimicrobials (109). In patients with VAP caused by MDR *S. maltophilia*, the following treatment options are safe and efficacious: (1) doxycycline and aerosolized colistin, and (2) IV colistin (27, 110, 111). Despite the emergence of antimicrobial resistance, fluoroquinolone prophylaxis may be considered as an appealing option in recipients of HSCT. However, their usefulness in prophylaxis in allogeneic HSCT should be evaluated in randomized trials (112, 113).

### New and future therapeutic modalities

New therapeutic strategies and future targeted therapies for *S. maltophilia* infections include the use of antimicrobial peptides, cationic compounds, bacteriophages, nanoemulsions, plant oils, constituents of green tea as well as inhibitors of β-lactamases and efflux pumps (2, 17).

The targeted therapies include bacterial efflux pumps inhibitors (BEPIs), inhibitors of β-lactamases, inhibitors of bacterial quorum sensing, antimicrobial peptides, bioengineered bacteriophage therapy, monoclonal antibodies directed against *S. maltophilia*, and antibodies to surface polysaccharides, which mediate biofilm formation and that are expressed during infection (Table 6) (114–124). Several families of BEPIs have been described (116). BEPIs have variable mechanisms of actions that include increasing intracellular concentrations of antibiotics that are expelled by efflux pumps, restoration of drug susceptibility of resistant clinical isolates, reduction of capability of acquired additional resistance, and reduction of intrinsic bacterial resistance to antimicrobials (114–116).

## Prevention of *S. maltophilia* Infections

Prevention of *S. maltophilia* infections can be achieved by: (1) health education of health-care personnel to prevent transmission and spread of this opportunistic pathogen, (2) observation of aqueous-associated environment and regular cleaning and disinfection of surfaces of medical instruments, (3) reinforcement of hand washing hygiene, (4) avoidance of using hospital tap water for bathing or cleaning of wounds, (5) discarding residual antibiotic solutions and contaminated fluids, (6) regular maintenance of hospital equipment and replacement of defective and worn parts, and (7) control of antimicrobial consumption (2, 17). Limitation of antimicrobial resistance and restriction of spread of the organism can be achieved by: appropriate antimicrobial selection, surveillance systems, and effective infection control measures (28).

## Course and Prognosis of *S. maltophilia* Infections

Morbidity and mortality associated with severe *S. maltophilia* infections are high due to the following reasons: (1) the organism is inherently resistant to antimicrobials, (2) antimicrobial resistance increases when the patient is colonized with the organism or given broad-spectrum antibiotics, and (3) the debilitated status of individuals acquiring the infection (6).

Risk factors for *S. maltophilia* infection-associated mortality include: (1) severely immunocompromised hosts, particularly those with underlying HM and recipients of HSCT, (2) septic shock, (3) organ failure, (4) bacteremia, (5) lung involvement such as pneumonia or pulmonary hemorrhage, (6) extensively drug-resistant organism, (7) exposure to carbapenems, (8) thrombocytopenia, and (9) high acute physiology and chronic health evaluation (APACHE) score in patients admitted to the ICU (2, 5, 7–9, 18, 60, 125, 126).

In *S. maltophilia*-associated infections, successful outcome is associated with administration of antibiotics to which the organism is susceptible, removal of infected devices such as CVCs, recovery of bone marrow function, taking enough preventive and isolation measures, and having a high index of suspicion (2, 8).

## Conclusion

*Stenotrophomonas maltophilia* is a globally emerging pathogen that causes serious infectious complications in immunocompromised patients in particular. It also exhibits a wide range of drug resistance mechanisms and this complicates the management of infections caused by this GNB. It is essential to utilize the available antimicrobials appropriately, use novel agents to which the organism is susceptible, and to strictly apply infection control measures in order to decrease the incidence of infections caused by *S. maltophilia*.

## Conflict of Interest Statement

The authors declare that the research was conducted in the absence of any commercial or financial relationships that could be construed as a potential conflict of interest.
